# Treatment of early caries lesions using biomimetic self-assembling peptides – a clinical safety trial

**DOI:** 10.1038/sj.bdj.2013.741

**Published:** 2013-08-24

**Authors:** P. A. Brunton, R. P. W. Davies, J. L. Burke, A. Smith, A. Aggeli, S. J. Brookes, J. Kirkham

**Affiliations:** 1 Restorative Dentistry, Leeds Dental Institute, University of Leeds, Clarendon Way, Leeds, LS2 9LU; 2 Centre for Molecular Nanoscience, School of Chemistry, University of Leeds, Leeds, LS2 9JT; 3 Oral Surgery, Leeds Dental Institute, University of Leeds, Clarendon Way, Leeds, LS2 9LU; 4 York Health Economics Consortium, Market Square, University of York, Heslington, York, YO10 5NH; 5 Biomineralisation Research Group, Leeds Dental Institute, University of Leeds, Clarendon Way, Leeds, LS2 9LU

## Abstract

**Objective** We previously reported that a rationally designed biomimetic self-assembling peptide, P_11_−4, nucleated hydroxyapatite *de novo* and was apparently capable of *in situ* enamel regeneration following infiltration into caries-like lesions. Our present aim was to determine the safety and potential clinical efficacy of a single application of P_11_−4 on early enamel lesions.

**Materials and methods** Fifteen healthy adults with Class V 'white spot' lesions received a single application of P_11_−4. Adverse events and lesion appearances were recorded over 180 days.

**Results** Patients treated with P_11_−4 experienced a total of 11 adverse events during the study, of which two were possibly related to the protocol. Efficacy evaluation suggested that treatment with P_11_−4 significantly decreased lesion size (p = 0.02) after 30 days and shifted the apparent progression of the lesions from 'arrested/progressing' to 'remineralising' (p <0.001). A highly significant improvement in the global impression of change was recorded at day 30 compared with baseline (p <0.001).

**Conclusions** The results suggest that treatment of early caries lesions with P_11_−4 is safe, and that a single application is associated with significant enamel regeneration, presumably by promoting mineral deposition within the subsurface tissue.

## Introduction

We previously described the effect of application of an anionic self-assembling peptide, P_11_−4, on the remineralisation and demineralisation behaviour of caries-like lesions under simulated intra-oral conditions.^1^ Peptide treatment significantly increased net mineral gain due to a combined effect of increased mineral gain and inhibition of mineral loss. In addition, P_11_−4 in its assembled form was shown to induce hydroxyapatite nucleation *de novo*. Based upon these data, we predicted that self-assembling peptides would be useful in the modulation of mineral behaviour during *in situ* dental tissue engineering.

P_11_−4 is a rationally-designed self-assembling peptide.^2^,^3^ Self-assembling peptides undergo well-characterised hierarchial self-assembly into three-dimensional fibrillar scaffolds in response to specific environmental triggers,^4^,^5^,^6^ offering a new generation of well-defined biopolymers with a range of potential applications.^7^ At peptide concentrations used here, P_11_−4 switches from a low viscosity isotropic liquid to an elastomeric nematic gel at pH <7.4 and in the presence of cations, conditions presumed to be found within a caries lesion.^8^ In a number of *in vitro* and *in vivo* experiments, the assembled P_11_−4 fibres were shown to be highly biocompatible with low immunogenicity.^9^,^10^

Assembled P_11_−4 forms scaffold-like structures with negative charge domains, mirroring biological macromolecules in mineralised tissue extracellular matrices (ECM).^11^ Proteins of the developing enamel ECM – themselves known to form self-assembling supramolecular structures^12^– have long been thought to control initial mineral deposition ('nucleation') and subsequent crystal growth, ultimately determining the physico-mechanical properties of the mature tissue.^13^ Mutations in enamel extracellular matrix proteins result in enamel defects in amelogenesis imperfecta, underlining the importance of protein-mineral interactions during enamel biomineralisation.^14^,^15^ Nucleation of mineral *de novo* is the first step in both the biomineralisation process and when designing biomaterials for mineralised tissue regeneration. Dental enamel mineral is comprised of a substituted hydroxyapatite, Ca_10_(PO_4_)_6_(OH)_2_ (Fig. 1a), with carbonate, fluoride and magnesium being the most common heteroionic substitutents.^16^ Nucleators act to bring together constituent mineral ions from the surrounding disorganised ionic mileu within the tissue fluids into a highly ordered crystal lattice structure by stabilising critical nucleii to permit crystal growth. In order to control the deposition of hydroxyapatite crystals during biomineralisation, critical ionic nuclei need to be formed by collision of relevant ions. Such nuclei need to be stable before subsequent crystal growth (Fig. 1b). Proteins of the extracellular matrices of mineralised tissues, which can bind constituent ions and stabilise critical nuclei, thus reducing the activation energy required ('heterogeneous nucleation') are important in the biological control of biomineralisation.

In a biomimetic strategy based on recapitulating normal enamel histogenesis, we proposed that following P_11_−4 self-assembly, the anionic groups of the P_11_−4 side chains would attract Ca^++^ ions, inducing *de novo* precipitation of hydroxyapatite as indicated in our earlier study.^1^

Enamel caries is a progressive subsurface demineralisation ultimately resulting in mechanical failure and cavitation.^17^ Invasive surgical treatment for enamel restoration has changed little in the past decades, despite advances in dental materials themselves. The earliest clinical sign of enamel caries is the appearance of a 'white spot' lesion on the tooth surface.^18^ At this stage, clinicians frequently elect to monitor lesion appearance, perhaps after the use of topical fluorides, to determine whether or not the lesion will progress, in which case a restoration would then be placed. Non-surgical intervention promoting defect biomineralisation or regeneration at the white spot lesion stage would remove the need to 'wait and see' and avoid the ultimate excavation of the tooth to place a restoration.

**Figure 2:**
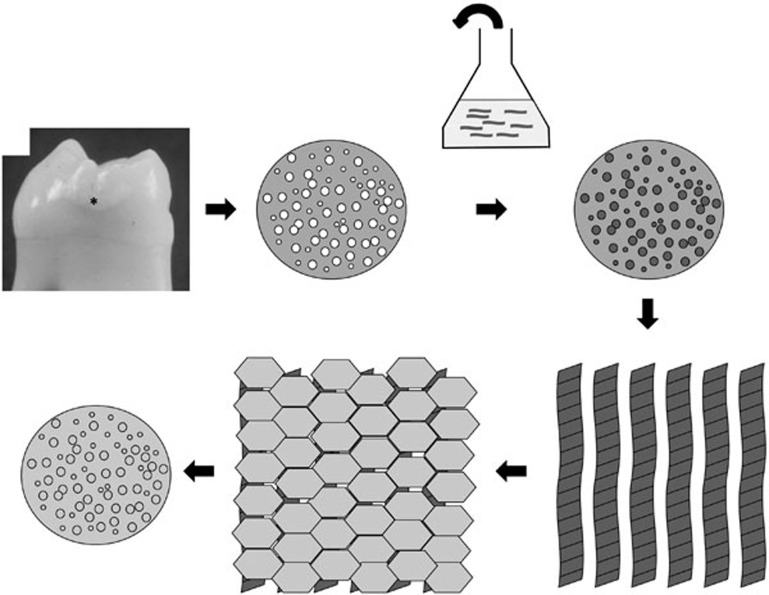
Schematic showing underlying hypothesis for treatment of early caries using P_11_−4 self assembling peptides. (1) Early caries appears as a 'white spot' on the enamel surface (^*^), this is due to the underlying porosity in the tissue (2). Aqueous P_11_−4 in its monomeric form applied to the lesion surface (3) will penetrate in to the pores due to its low viscosity (4). Self-assembly is triggered by the conditions (pH <7.4, presence of salts) within the lesion forming fibres (5) which can nucleate hydroxyapatite mineral (6). This restores both the enamel mineral and the natural appearance (7) of the original lesion

We hypothesised that infiltration of early ('white spot') caries lesions using low viscosity monomeric P_11_−4 would result in triggered self-assembly within the lesion, generating a subsurface bioactive scaffold capable of recapitulating normal histogenesis by inducing mineral deposition *in situ* (Fig. 2).

The specific aim of the present study was to conduct a first-in-man clinical safety study applying a single treatment of P_11_−4 to Class V enamel lesions in healthy human volunteers.

## Materials and methods

### Preparation of P_11_−4

HPLC purified peptide P_11_−4 (CH_3_CO-QQRFEWEFEQQ-CONH_2_) was acquired from PolyPeptide Group (France). Mass spectrometry found an experimental molecular weight of 1,595.8 (expected molecular weight = 1,595.7). Peptide content was determined by amino acid analysis and UV absorbance and found to be 89.2 ± 2.5% (expected maximum peptide content = 90.5%, calculated taking into account only bound counterions, not bound water).

P_11_−4 was dissolved in water (18.2 MΩ-cm) at a concentration of 6.3 mM. A solution of 1% ammonia was added drop wise until a pH of 8.6 ± 0.1 was achieved. The sample was then vortexed and sonicated to produce a clear colourless monomeric fluid. Aliquots (0.05 ml) of monomeric P_11_−4 were pipetted into individual vials, flash frozen with liquid nitrogen and lyophilised for 24 hours. The individual samples were then sealed with a crimp.

### Selection of volunteers

Ethical approval was obtained through the UK National Research Ethics System (NRES; project number 10/H1207/75). Subjects were recruited from staff and patients attending Leeds Dental Institute (LDI). The study was conducted according to ISO 14155:2003 in the University of Leeds Translational and Clinical Research Unit at LDI. Fifteen subjects with 19 lesions were recruited according to the following criteria: included in the study were:

- Subjects with ≥1 visible and accessible early caries lesion present, which did not require operative intervention
- Subjects aged between 18 and 65 years who were willing and able to observe good oral hygiene and attend for the study visits
- Subjects with more than 20 teeth present and a Basic Periodontal Examination (BPE) score of less than three
- Subjects who were willing and able to understand all study-related procedures.

Excluded from the study were:

- Subjects with >1 caries lesion present on the selected tooth
- Subjects who had a significant medical history, including pregnancy and breast feeding or if they smoked more than five cigarettes per day
- Subjects who had evidence of reduced salivary flow or significant tooth wear
- Subjects who were participating in concurrent clinical trials or who had recently participated in other clinical trials of an investigational drug or device.

Mean subject age for the study group was 34.4 ± 12.7 years. The group comprised of seven females and eight males. Tooth type, lesion position and lesion activity are shown in Table 1.

### Baseline assessment (Day 1)

The following baseline assessments were performed:

- A general oral and physical examination including a dental, medical and social history and vital signs
- A series of digital colour photographs of the test lesion taken under standard conditions of light and magnification
- A detailed relevant medical (including dental) history covering the previous three years and any medication they were taking at the time of the study.

At each recall the patients were questioned to determine whether or not they might have experienced any adverse events since their last visit and to identify any new medication(s) that they had taken since joining the trial.

### Clinical procedures

The test lesion was cleaned with a prophylaxis paste (Kemdent Flour of Pumice, ADP Ltd, Swindon, UK) and isolated. The lesion was then treated with etch solution (Super Etch, SDI Ltd, Bayswater, Victoria, Australia) for 30 seconds to open up the pores to the subsurface lesion and subsequently washed and dried. Lyophilised P_11_−4 was rehydrated with 0.05 ml of sterile water and a single drop of the resulting solution immediately applied directly to the lesion surface. Moisture control was ensured until the P_11_−4 solution was no longer visible (approximately two minutes).

Subjects were advised not to brush the quadrant containing the treated tooth until Day 4 (D4) review visit. Instead, subjects were asked to rinse that area of the mouth with a chlorhexidine mouthwash (Corsodyl, GSK, Herrenberg, Germany). At D4, a soft toothbrush and toothpaste were provided and subjects asked to use these until Day 8 (D8) review visit. At this point they reverted to their normal oral hygiene procedures.

### Review assessments (D4, D8, D30 and D180)

Subjects were reviewed at 4, 8, 30 and 180 days after treatment. At each visit the following assessments were conducted:

- Visual inspection of the treated site was carried out and any potential adverse events recorded
- Vital signs were recorded
- A series of colour photographs of the test lesion were taken under standard conditions of light and magnification
- At D30 and D180, a Global Impression of Change questionnaire was completed by both the subjects and dental professionals.

### Assessment of lesion appearance and progression

Colour photographs of all lesions from all subjects were used to assess lesion size, appearance and progression. Blind assessment was carried out independently by two experienced clinicians using a global impression of change questionnaire and a visual analogue scale (VAS).

Interim assessment was carried out by comparing lesion size and appearance at D4, D8 and D30 to D1 (baseline). Final assessment was carried out by comparing D180 with D30 and correlating the data to the interim assessment.

Colour photographs for all assessments were selected by a member of staff who was not involved in the study and were each allocated a unique identifier including the day of the visit in order that comparisons with baseline might be made. As such, the assessors would not have been blind to the identity of the lesions at baseline. To address this, D1 and D30 photographs were 'switched' according to a randomisation table such that the assessors did not know whether they were making comparisons against 'true' D1 baseline. Neither assessor was aware which baseline data had been 'switched'. Where 'switching' had occurred, a real improvement with time after treatment would appear as an apparent deterioration.

Unblinding was performed after completion of all assessments and database lock.

### Image analysis

For each lesion, digital images were selected from photographs taken at baseline (D1), D30 and D180. Lesion contour was marked by a blinded operator and the total number of pixels within contour boundaries determined using image analysis (Soft Imaging System, Münster, Germany). Only those images where tooth orientation was consistent across the three visits were used (validated using tooth landmarks and a series of interconnecting lines) and hence a total of 12 (D1 & D30) and 11 (D180) lesions were analysed.

### Statistical analysis

Data was analysed by an independent statistician at the University of York.

Independent sample t-tests were used to determine any statistically significant differences between the two assessors' ratings for colour, size, progression and global impression of change.

A repeated measures t-test was used to assess statistically significant changes over time in the colour, size, progression assessments and the global impression of change. Fisher's method was used to combine p-values from the repeated measures t-tests for the two assessors, producing chi-squared test statistics that can be evaluated for statistical significance against 2k degrees of freedom for k number of tests. The alpha value was adjusted to compensate for positively associated tests (that is, lack of independence) using a(k + 1)/(2k),providing an alpha value of 0.0375.

## Results

All subjects completed all stages of the study with the exception of one individual who was lost to follow-up at D180 due to their relocation outside of the area. There were 11 recorded adverse events (See Table 2) of which two were judged by the investigator as probably related to the protocol used in the trial. One was a transient dental hypersensitivity; the other a sensitivity to the Corsodyl mouthwash provided within the study. All other adverse events were judged to be not related to the study. None of the adverse events were classified as serious.

**Figure 3:**
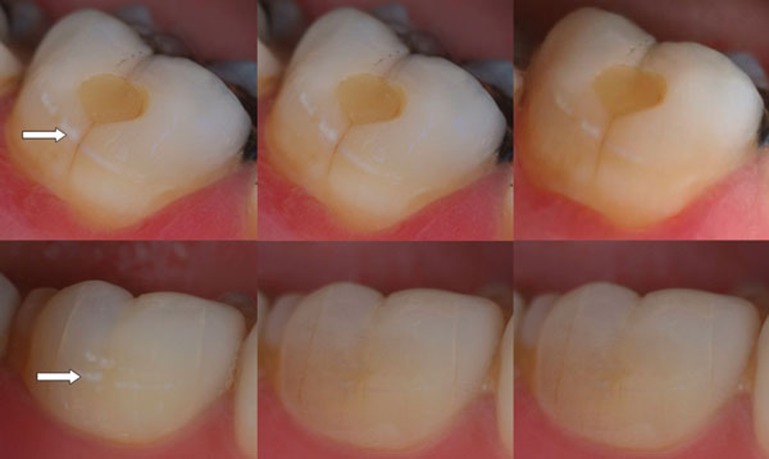
Examples of clinical appearance of two Class V caries lesions (arrows) in different subjects included in the study. Images selected to show the range of response to a single application of P_11_−4 with time. (a, d) Lesion appearance at baseline (D0); (b, e) and (c, f) appearance of same lesion at D30 and D180 respectively

The effect of treating the lesions using P_11_−4 was initially assessed based upon their clinical appearance using clinical photographs (Fig. 3). Statistical analysis of the scoring by the two assessors showed no significant differences in their assessments across all of the measured outcomes.

**Figure 4:**
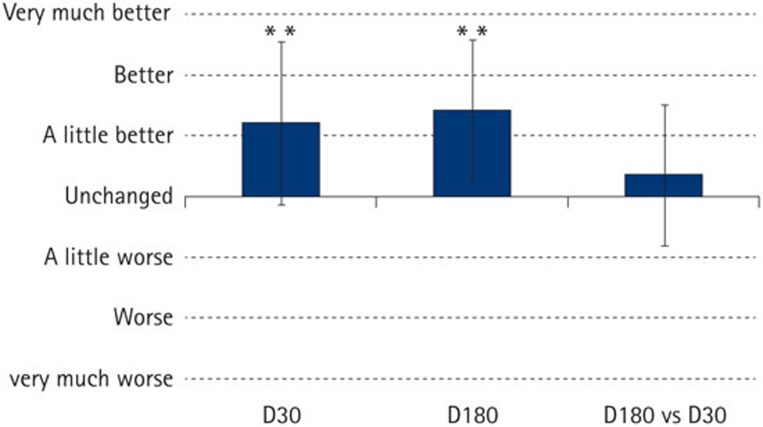
Schematic showing underlying hypothesis for treatment of early caries using P_11_−4 self assembling peptides. (1) Early caries appears as a 'white spot' on the enamel surface (^*^), this is due to the underlying porosity in the tissue (2). Aqueous P_11_−4 in its monomeric form applied to the lesion surface (3) will penetrate in to the pores due to its low viscosity (4). Self-assembly is triggered by the conditions (pH <7.4, presence of salts) within the lesion forming fibres (5) which can nucleate hydroxyapatite mineral (6). This restores both the enamel mineral and the natural appearance (7) of the original lesion

The results of the clinicians' global impression of change questionnaire are shown in Figure 4. Compared with baseline, there was a significant improvement in the assessment scores 30 days after a single application of P_11_−4 (p <0.001). This improvement was maintained even after 180 days post-treatment; results compared with baseline showed a highly significant improvement in the global impression of change scores (p <0.0001). No statistically significant differences in the global impression of change scores were seen when D180 was compared with D30, suggesting that the major treatment effect for P_11_−4 had occurred by D30 post-treatment.

A VAS was used to assess specific lesion parameters including colour, size and progression based upon the appearance of the lesion in the clinical photographs.

**Figure 5:**
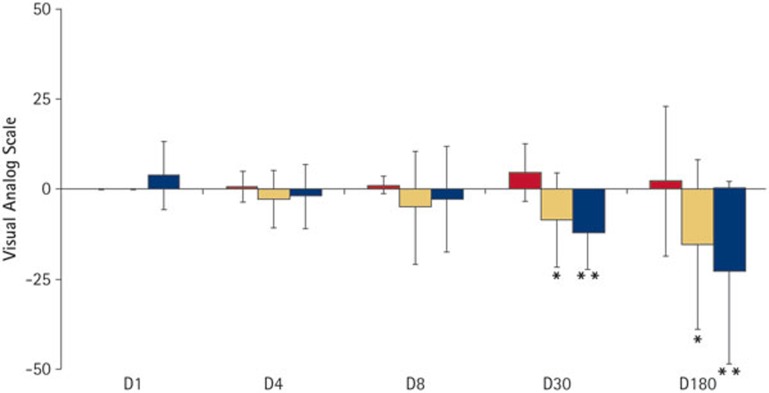
Effect of a single application of P_11_−4 on colour, size and progression of the treated caries lesion compared to baseline (D0) as judged by blinded assessors using a visual analogue scale from −100 to +100. Histograms show change in lesion colour (red); change in lesion size (yellow) and change in lesion progression (blue). Lesion colour: positive values indicate an improvement in lesion colour (ie less white). Lesion size: positive values indicate a lesion that is increasing in size; negative values a decrease in lesion size. Lesion progression: positive values indicate a lesion that is judged to be progressing, negative values indicate a lesion that is judged to be remineralising. Significance when compared with baseline indicated by ^*^(p ≤0.05); ^**^(p ≤0.001)

Figure 5 summarises the VAS data for each of the follow up visits compared with. baseline. No significant differences were seen in any of the three parameters in the first follow up visit (D4). By D8, there was a significant difference observed in lesion colour (p <0.05) but this was not significant at any of the later time points, mainly due to the very large variation in the scoring. For lesion size and progression, there was an increasing trend towards improvement over the first eight days after treatment but this was not statistically significant until D30, where lesion size was judged to have significantly decreased (p <0.05) and lesion progression was judged to have moved from arrested/progressing to remineralising (p <0.01). This improvement in lesion size and progression was maintained at D180 (almost six months after the single application of P_11_−4).

Finally, in agreement with the clinical assessment, morphometic analysis also showed a significant decrease in lesion size compared with baseline at D30 (31 ± 24%, n = 11; p = 0.002) and D180 (40 ± 27%, n = 11; p = 0.001).

## Discussion

This small study represents a first-in-man clinical safety trial of a regenerative caries therapeutic that has been rationally designed to recapitulate the processes occurring during enamel biomineralisation, where a self-assembled organic matrix controls the deposition and growth of hydroxyapatite crystals.^13^ As a potential treatment for early caries lesions, P_11_−4 is safe, non-invasive and acceptable to patients (as evidenced by the unanimous and significantly positive response on the patients' global impression of change questionnaire [p = 0.0003]). However, it is important to note that this is not an efficacy trial and as such we cannot compare the data obtained here with a 'do nothing' control or any other intervention, such as the application of topical fluoride.

We have previously shown that P_11_−4 is able to nucleate hydroxyapatite *de novo* and is able to promote repair of caries-like lesions *in vitro*.^1^ The treatment differs from other 'filling without drilling' infiltrative strategies in that P_11_−4 is a bioactive peptide synthesised from natural amino acids that is triggered to assemble into a three dimensional fibrillar scaffold by environmental conditions of pH and salt concentration. Assembly takes place within the lesion, and the scaffold can then act as nucleator for hydroxyapatite, effecting tissue regeneration from within. Infiltration of lesion pores using low viscosity resins such as sealants has been demonstrated to halt caries progression *in vitro*,^19^,^20^ *in situ*^21^ and *in vivo*,^22^ presumably by providing a barrier to diffusion within the lesion. The use of a biomimetic peptide such as P_11_−4 has the additional advantage of effecting 'natural' repair by regenerating the mineral itself.

We used a global impression of change questionnaire coupled with assessment of clinical photographs using VAS and morphometric analysis of lesion size to determine any clinical benefit following treatment of the lesions with P_11_−4. Visual analogue scales are commonly used in dental research, for example in judging severity of fluorosis.^23^ We chose not to use radiographs as an outcome measure as the changes we were seeking to identify are relatively small, given that these are early lesions which do not themselves necessarily show on radiographs. We chose not to use caries detection devices as those currently available depend on surrogate indicators of lesion progression/detection (eg QLF depends on the enamel structure; Diagnodent depends on presence of porphyrins; CarieScan depends on conductivity or water) though it would be interesting to utilise these diagnostics in future efficacy trials. The results of the study indicate that the clinical use of P_11_−4 is safe and, based upon clinical judgement, Class V lesions treated with a single application of P_11_−4 show significant improvement in their clinical appearance by D30. This improvement in size and progression was maintained even after 180 days post-treatment and was further supported by the morphometric analysis. We do not know the kinetics of peptide-induced remineralisation but the data suggests a safe and positive trend beginning at the earliest days post-treatment. It is also possible that the potential for lesion repair might be influenced by the initial state of the lesion itself. We have no historical data relating to those lesions that were treated here and the small sample size means that we cannot make any evaluation as to whether initial lesion activity is related to treatment outcome. This would need to be addressed as part of a more comprehensive efficacy trial.

Despite the fact that this study was a small, non-controlled safety clinical trial, all of the outcome measures used showed beneficial results in respect of enamel regeneration. The results are therefore promising given that we have not yet investigated the optimum clinical delivery for P_11_−4 or the formulation of P_11_−4 itself. It may be that we can improve efficacy by pre-treating lesions to remove extraneous material, such as proteins that may be present within.^24^ In addition, we have yet to determine whether repeated application of P_11_−4 would provide further enamel benefit for those lesions where repair was incomplete. Given that P_11_−4 is a well-tolerated treatment, repeated application is an obvious way forward. Finally, we are currently designing and bench testing 'next generation' peptides to accelerate the repair process, making 'filling without drilling' a reality.

## Figures and Tables

**Figure 1:**
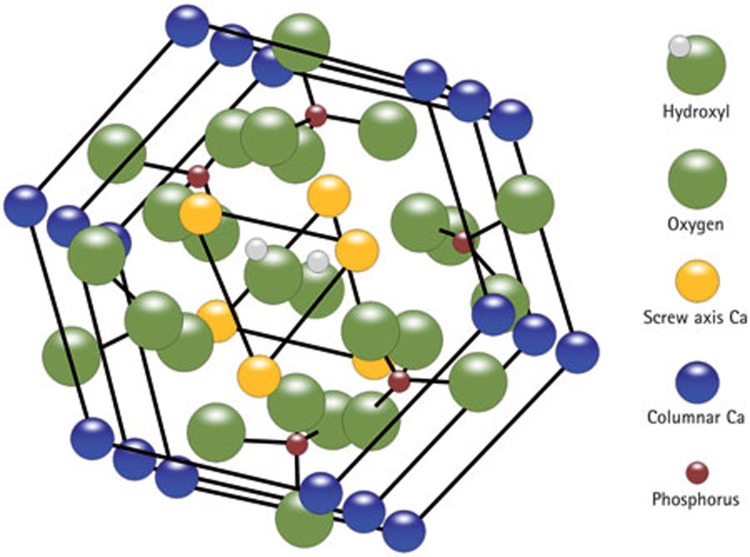
Diagrammatic illustration of structure of hydroxyapatite, originally described by Kay *et al*.,^25^ and modified here from Elliott^26^

**Figure 1:**
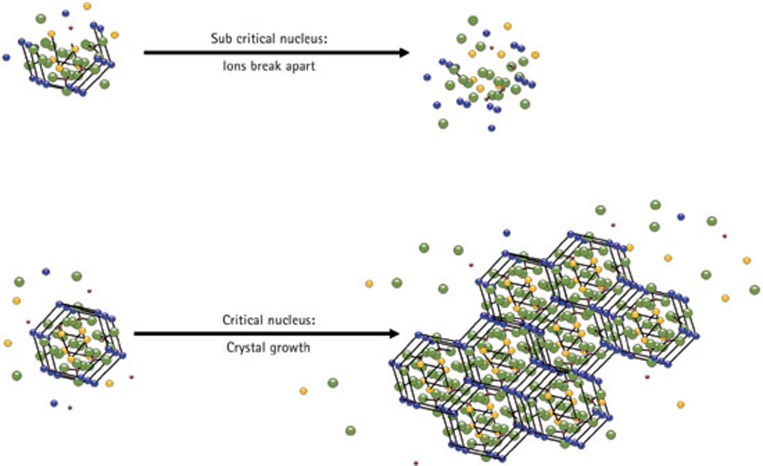
Diagram illustrating nucleation and crystal growth

**Table 1:** Tooth type, lesion position and lesion activity at baseline for teeth treated with P_11_−4

Patient-number	Tooth number and lesion position*	Lesion activity at baseline as judged by VAS**
1-001	36b	−0.5
1-002	46b	20.5
1-003	46b	11
1-004	46b	−11
1-005	46b	−5.5
1-006	46b	−3.5
1-007	46b	6
1-008	46b	−2.5
1-009	16b	1.5
1-010	16l	15
1-011	44b	7
1-012	35b	3.5
1-013	43b	9.5
1-014	14b	9.5
1-015	24b	−16.5

^*^b = buccal; l = lingual

^**^VAS scale from −50 to +50; negative numbers represent a remineralising lesion; positive numbers present an active lesion. Arrested lesions score '0'.

**Table 2:** Recorded adverse events

Patient number	Adverse event	Treatment causality
1-002	Chest infection	Not related
1-003	Dental hypersensitivity	Possibly related
1-003	Sprained joint between spine and pelvis	Not related
1-004	Stomach upset	Not related
1-005	Sensitivity with corsodyl mouthrinse	Possibly related
1-006	Diarrhoea and vomiting	Not related
1-007	Superficial staining	Not related
1-012	Chest infection	Not related
1-013	Dental treatment	Not related
1-015	Operation on Dupuytren's Contraction	Not related
1-015	Routine dental treatment	Not related

## Notes

**Refereed Paper**

## References

[b1] Kirkham J, Firth A, Vernals D . Self-assembling peptide scaffolds promote enamel remineralization. J Dent Res 2007; 86: 426–430. 17452562 10.1177/154405910708600507

[b2] Aggeli A, Bell M, Boden N . Responsive gels formed by the spontaneous self-assembly of peptides into polymeric beta-sheet tapes. Nature 1997; 386: 259–262. 9069283 10.1038/386259a0

[b3] Aggeli A, Bell M, Boden N . Engineering of peptide beta-sheet nanotapes. J Mater Chem 1997; 7: 1135–1145.

[b4] Aggeli A, Bell M, Carrick L M . pH as a trigger of peptide beta-sheet self-assembly and reversible switching between nematic and isotropic phases. J Am Chem Soc 2003; 125: 9619–9628. 12904028 10.1021/ja021047i

[b5] Aggeli A, Fytas G, Vlassopoulos D . Structure and dynamics of self-assembling beta-sheet peptide tapes by dynamic light scattering. Biomacromolecules 2001; 2: 378–388. 11749196 10.1021/bm000080z

[b6] Aggeli A, Nyrkova I A, Bell M . Hierarchical self-assembly of chiral rod-like molecules as a model for peptide beta -sheet tapes, ribbons, fibrils, and fibres. Proc Natl Acad Sci U S A 2001; 98: 11857–11862. 11592996 10.1073/pnas.191250198 PMC59814

[b7] Firth A, Aggeli A, Burke J L, Yang X, Kirkham J. Biomimetic self-assembling peptides as injectable scaffolds for hard tissue engineering. Nanomedicine (Lond) 2006; 1: 189–199. 17716108 10.2217/17435889.1.2.189

[b8] Kitasako Y, Hiraishi N, Nakajima M . *In vitro* surface analysis of active and arrested dentinal caries using a pH-imaging microscope. Oper Dent 2002; 27: 354–359. 12120772

[b9] Burke J L. In situ engineering of skeletal tissues using self-assembled biomimetic scaffolds [PhD]. Leeds: The University of Leeds, 2011.

[b10] Felton S. Self assembling -sheet peptide networks as smart scaffolds for tissue engineering [PhD Thesis]. Leeds: University of Leeds, 2005.

[b11] Kirkham J, Brookes S J, Shore R C . Physico-chemical properties of crystal surfaces in matrix- mineral interactions during mammalian biomineralisation. Curr Opin Colloid In 2002; 7: 124–132.

[b12] Wen H B, Moradian-Oldak J, Leung W, Bringas P Jr., Fincham A G. Microstructures of an amelogenin gel matrix. J Struct Biol 1999; 126: 42–51. 10329487 10.1006/jsbi.1999.4086

[b13] Simmer J P, Fincham A G. Molecular mechanisms of dental enamel formation. Crit Rev Oral Biol M 1995; 6: 84–108. 7548623 10.1177/10454411950060020701

[b14] Kim J W, Seymen F, Lin B P . ENAM mutations in autosomal-dominant amelogenesis imperfecta. J Dent Res 2005; 84: 278–282. 15723871 10.1177/154405910508400314

[b15] Wright J T, Hart P S, Aldred M J . Relationship of phenotype and genotype in X-linked amelogenesis imperfecta. Connect Tissue Res 2003; 44: 72–78. 12952177

[b16] Robinson C, Kirkham J, Brookes S J, Shore R C. Chemistry of mature enamel. *In* Robinson C, Kirkham J, Shore R C (eds) Dental enamel formation to destruction. pp 167–191. Boca Raton: CRC Press, 1995.

[b17] Robinson C, Shore R C, Brookes S J . The chemistry of enamel caries. Crit Rev Oral Biol M 2000; 11: 481–495. 11132767 10.1177/10454411000110040601

[b18] Johnson A R. The early carious lesion of enamel. J Oral Pathol 1975; 4: 128–157. 810551 10.1111/j.1600-0714.1975.tb01861.x

[b19] Fan PL, Seluk L W, O'Brien W J. Penetrativity of sealants: I. J Dent Res 1975; 54: 262–264. 1054336

[b20] Robinson C, Brookes S J, Kirkham J, Wood S R, Shore R C. *In vitro* studies of the penetration of adhesive resins into artificial caries-like lesions. Caries Res 2001; 35: 136–141. 11275674 10.1159/000047445

[b21] Paris S, Meyer-Lueckel H. Inhibition of caries progression by resin infiltration *in situ*. Caries Res 2010; 44: 47–54. 20090328 10.1159/000275917

[b22] Paris S, Hopfenmuller W, Meyer-Lueckel H. Resin infiltration of caries lesions: an efficacy randomized trial. J Dent Res 2010; 89: 823–826. 20505049 10.1177/0022034510369289

[b23] Vieira A P, Lawrence H P, Limeback H, Sampaio F C, Grynpas M. A visual analogue scale for measuring dental fluorosis severity. J Am Dent Assoc 2005; 136: 895–901. 16060470 10.14219/jada.archive.2005.0290

[b24] Shore R C, Kirkham J, Brookes S J, Wood S R, Robinson C. Distribution of exogenous proteins in caries lesions in relation to the pattern of demineralisation. Caries Res 2000; 34: 188–193. 10773638 10.1159/000016588

[b25] Kay MI, Young R A, Posner A S. Crystal structure of hydroxyapatite. Nature 1964; 204: 1050–1052. 14243377 10.1038/2041050a0

[b26] Elliott J C. Structure and chemistry of the apatites and other calcium orthophosphates. Amsterdam: Elsevier, 1994.

